# Bis(ethyl­enediamine-κ^2^ *N*,*N*′)bis­(phenytoinato-κ*N*)cobalt(II)

**DOI:** 10.1107/S1600536809044092

**Published:** 2009-10-31

**Authors:** Xi-Lan Hu, Xing-You Xu, Da-Qi Wang, Yan-Qin Zhou

**Affiliations:** a Huaihai Institute of Technology, Jiangsu 222005, People’s Republic of China; b Huaiyin Institute of Technology, Jiangsu 223003, People’s Republic of China; c College of Chemistry and Chemical Engineering, Liaocheng University, Shandong 252059, People’s Republic of China

## Abstract

The title compound [systematic name: bis(2,5-dioxo-4,4-diphenylimidazolidin-1-ido-κ*N* ^1^)bis(ethylenediamine-κ^2^ *N*,*N*′)cobalt(II)], [Co(C_15_H_11_N_2_O_2_)_2_(C_2_H_8_N_2_)_2_], has site symmetry 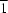. The Co^II^ cation is located on an inversion center and coordinated by two phenytoin anions and two ethyl­enediamine ligands in a distorted octa­hedral geometry. In the phenytoin anion, the two phenyl rings are twisted with respect to the central hydantoin ring, making dihedral angles of 77.49 (16) and 64.55 (15)°. Intra­molecular and inter­molecular N—H⋯O hydrogen bonding is present in the crystal structure.

## Related literature

For applications of phenytoin, see: Akitsu & Einaga (2005 ▶); Akitsu *et al.* (1997 ▶). For related compounds, see: Hu *et al.* (2006 ▶, 2007 ▶).
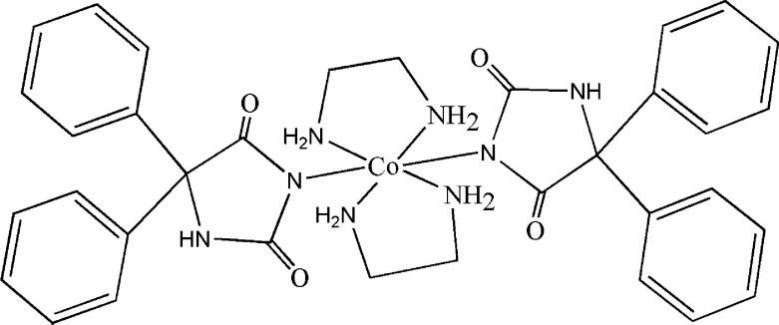


## Experimental

### 

#### Crystal data

[Co(C_15_H_11_N_2_O_2_)_2_(C_2_H_8_N_2_)_2_]
*M* *_r_* = 681.65
Monoclinic, 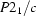

*a* = 11.8035 (12) Å
*b* = 12.3439 (13) Å
*c* = 11.0768 (10) Å
β = 92.277 (1)°
*V* = 1612.6 (3) Å^3^
*Z* = 2
Mo *K*α radiation
μ = 0.58 mm^−1^
*T* = 298 K
0.52 × 0.42 × 0.28 mm

#### Data collection

Bruker SMART CCD area-detector diffractometer
Absorption correction: multi-scan (*SADABS*; Sheldrick, 1996 ▶) *T*_min_ = 0.751, *T*_max_ = 0.854
7877 measured reflections
2836 independent reflections
2201 reflections with *I* > 2σ(*I*)
*R*_int_ = 0.053

#### Refinement

*R*[*F* ^2^ > 2σ(*F* ^2^)] = 0.043
*wR*(*F* ^2^) = 0.120
*S* = 1.08
2836 reflections
214 parameters
H-atom parameters constrained
Δρ_max_ = 0.45 e Å^−3^
Δρ_min_ = −0.63 e Å^−3^

### 

Data collection: *SMART* (Siemens, 1996 ▶); cell refinement: *SAINT* (Siemens, 1996 ▶); data reduction: *SAINT*; program(s) used to solve structure: *SHELXTL* (Sheldrick, 2008 ▶); program(s) used to refine structure: *SHELXTL*; molecular graphics: *SHELXTL*; software used to prepare material for publication: *SHELXTL*.

## Supplementary Material

Crystal structure: contains datablocks I, global. DOI: 10.1107/S1600536809044092/xu2649sup1.cif

Structure factors: contains datablocks I. DOI: 10.1107/S1600536809044092/xu2649Isup2.hkl

Additional supplementary materials:  crystallographic information; 3D view; checkCIF report

## Figures and Tables

**Table 1:** Selected bond lengths (Å)

Co1—N2	2.180 (2)
Co1—N3	2.123 (2)
Co1—N4	2.171 (2)

**Table 2:** Hydrogen-bond geometry (Å, °)

*D*—H⋯*A*	*D*—H	H⋯*A*	*D*⋯*A*	*D*—H⋯*A*
N1—H1⋯O2^i^	0.86	2.00	2.861 (3)	173
N3—H3*A*⋯O1^ii^	0.90	2.18	2.947 (3)	143
N3—H3*B*⋯O2	0.90	2.20	2.966 (3)	143

Symmetry codes: (i) 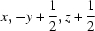; (ii) 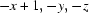.

**Fig. 1.**
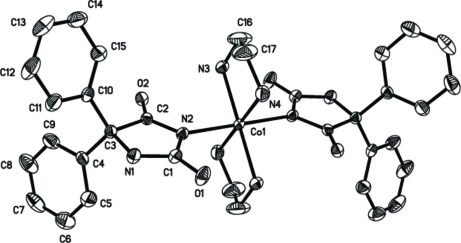
The molecular structure of the title complex. Displacement ellipsoids are drawn at the 30% probability level. The H-atom have been omitted for clarity.

## supplementary crystallographic information

### Comment

5,5-Diphenylimidazoline-2,4-dione (phenytoin) compound is a widely used drug in the treatment of epilepsy and should be an excellent ligand for transition metal complex (Akitsu *et al.*,  1997; Akitsu & Einaga, 2005). We have synthesized a series of complexes with 5,5-diphenylhydantoinate ligand (Hu *et al.*, 2006, 2007). We report here the crystal structure of the title compound.

The compound (Fig. 1) consists of [Co(pht)_2_(en)_2_] (Hpht = 5,5-diphenylhydantoin; en = ethylendiamine) complex neutral molecule. The Co atom is coordinated by two nitrogen atoms from two Hpht ligands and four nitrogen atoms from two en ligands in a distorted octahedral CoN_6_ coordination environment (Table 1). The Co—N bond distances lie in the range of 2.123 (2) Å to 2.180 (2) Å. There are intra- and intermolecular N—H···O hydrogen bonds (Table 2).

### Experimental

To a solution of Hpht (1 mmol) in methanol (10 ml) was added cobalt acetate tetrahydrate (0.5 mmol) and the solution of ethylenediamine (1 mmol) in methanol (10 ml). Then the mixture was sealed in a 25 ml stainless steel vessel with Teflon linear, and heated at 393 K for 50 h. After cooling to room temperature, the orange single crystals were obtained.

### Refinement

H atoms were placed at calculated positions with N—H = 0.86–0.90 Å and C—H = 0.93–0.97 Å, and refined in riding mode with U_iso_(H) = 1.2U_eq_(C,N).

### Crystal data

**Table:**

[Co(C_15_H_11_N_2_O_2_)_2_(C_2_H_8_N_2_)_2_]	*F*(000) = 714
*M**_r_* = 681.65	*D*_x_ = 1.404 Mg m^−^^3^
Monoclinic, *P*2_1_/*c*	Mo *K*α radiation, λ = 0.71073 Å
Hall symbol: -P 2ybc	Cell parameters from 3389 reflections
*a* = 11.8035 (12) Å	θ = 2.4–26.9°
*b* = 12.3439 (13) Å	µ = 0.58 mm^−^^1^
*c* = 11.0768 (10) Å	*T* = 298 K
β = 92.277 (1)°	Block, orange
*V* = 1612.6 (3) Å^3^	0.52 × 0.42 × 0.28 mm
*Z* = 2	

### Data collection

**Table:**

Bruker SMART CCD area-detector diffractometer	2836 independent reflections
Radiation source: fine-focus sealed tube	2201 reflections with *I* > 2σ(*I*)
graphite	*R*_int_ = 0.053
φ and ω scans	θ_max_ = 25.0°, θ_min_ = 1.7°
Absorption correction: multi-scan (*SADABS*; Sheldrick, 1996)	*h* = −14→11
*T*_min_ = 0.751, *T*_max_ = 0.854	*k* = −14→14
7877 measured reflections	*l* = −13→12

### Refinement

**Table:**

Refinement on *F*^2^	Primary atom site location: structure-invariant direct methods
Least-squares matrix: full	Secondary atom site location: difference Fourier map
*R*[*F*^2^ > 2σ(*F*^2^)] = 0.043	Hydrogen site location: inferred from neighbouring sites
*wR*(*F*^2^) = 0.120	H-atom parameters constrained
*S* = 1.08	*w* = 1/[σ^2^(*F*_o_^2^) + (0.0528*P*)^2^ + 0.9459*P*] where *P* = (*F*_o_^2^ + 2*F*_c_^2^)/3
2836 reflections	(Δ/σ)_max_ = 0.001
214 parameters	Δρ_max_ = 0.45 e Å^−^^3^
0 restraints	Δρ_min_ = −0.62 e Å^−^^3^

### Special details

**Table:**

Geometry. All esds (except the esd in the dihedral angle between two l.s. planes) are estimated using the full covariance matrix. The cell esds are taken into account individually in the estimation of esds in distances, angles and torsion angles; correlations between esds in cell parameters are only used when they are defined by crystal symmetry. An approximate (isotropic) treatment of cell esds is used for estimating esds involving l.s. planes.
Refinement. Refinement of F^2^ against ALL reflections. The weighted R-factor wR and goodness of fit S are based on F^2^, conventional R-factors R are based on F, with F set to zero for negative F^2^. The threshold expression of F^2^ > 2sigma(F^2^) is used only for calculating R-factors(gt) etc. and is not relevant to the choice of reflections for refinement. R-factors based on F^2^ are statistically about twice as large as those based on F, and R- factors based on ALL data will be even larger.

### Fractional atomic coordinates and isotropic or equivalent isotropic displacement parameters (Å^2^)

**Table:**

	*x*	*y*	*z*	*U*_iso_*/*U*_eq_	
Co1	0.5000	0.0000	0.0000	0.02790 (18)	
N1	0.69362 (19)	0.2364 (2)	0.21879 (19)	0.0366 (6)	
H1	0.7028	0.2732	0.2843	0.044*	
N2	0.61498 (18)	0.11950 (18)	0.08239 (18)	0.0300 (5)	
N3	0.63769 (19)	−0.0802 (2)	−0.0794 (2)	0.0412 (6)	
H3A	0.6163	−0.1046	−0.1534	0.049*	
H3B	0.6959	−0.0339	−0.0869	0.049*	
N4	0.5465 (2)	−0.1072 (2)	0.1492 (2)	0.0437 (6)	
H4A	0.5499	−0.0702	0.2193	0.052*	
H4B	0.4951	−0.1606	0.1549	0.052*	
O1	0.53186 (17)	0.14774 (19)	0.26695 (18)	0.0513 (6)	
O2	0.74262 (16)	0.13811 (15)	−0.06839 (15)	0.0335 (5)	
C1	0.6080 (2)	0.1661 (2)	0.1968 (2)	0.0336 (6)	
C2	0.7082 (2)	0.1602 (2)	0.0329 (2)	0.0277 (6)	
C3	0.7685 (2)	0.2423 (2)	0.1181 (2)	0.0304 (6)	
C4	0.7646 (2)	0.3540 (2)	0.0582 (2)	0.0353 (7)	
C5	0.6736 (3)	0.4219 (3)	0.0689 (4)	0.0608 (10)	
H5	0.6148	0.4013	0.1174	0.073*	
C6	0.6674 (4)	0.5196 (3)	0.0096 (5)	0.0799 (13)	
H6	0.6046	0.5642	0.0177	0.096*	
C7	0.7544 (4)	0.5513 (3)	−0.0617 (3)	0.0715 (12)	
H7	0.7504	0.6174	−0.1019	0.086*	
C8	0.8464 (4)	0.4856 (3)	−0.0734 (3)	0.0688 (12)	
H8	0.9051	0.5067	−0.1217	0.083*	
C9	0.8516 (3)	0.3875 (3)	−0.0129 (3)	0.0538 (9)	
H9	0.9148	0.3434	−0.0203	0.065*	
C10	0.8891 (2)	0.2084 (2)	0.1586 (2)	0.0329 (6)	
C11	0.9473 (3)	0.2721 (3)	0.2414 (3)	0.0527 (9)	
H11	0.9147	0.3362	0.2674	0.063*	
C12	1.0538 (3)	0.2423 (4)	0.2868 (3)	0.0681 (11)	
H12	1.0921	0.2864	0.3430	0.082*	
C13	1.1027 (3)	0.1489 (4)	0.2494 (3)	0.0681 (12)	
H13	1.1735	0.1282	0.2812	0.082*	
C14	1.0475 (3)	0.0858 (3)	0.1654 (3)	0.0618 (10)	
H14	1.0817	0.0231	0.1380	0.074*	
C15	0.9402 (3)	0.1147 (3)	0.1204 (3)	0.0473 (8)	
H15	0.9025	0.0705	0.0640	0.057*	
C16	0.6722 (4)	−0.1698 (4)	−0.0025 (4)	0.0868 (14)	
H16A	0.6294	−0.2335	−0.0277	0.104*	
H16B	0.7517	−0.1849	−0.0143	0.104*	
C17	0.6569 (4)	−0.1520 (4)	0.1242 (4)	0.0873 (15)	
H17A	0.7153	−0.1028	0.1551	0.105*	
H17B	0.6665	−0.2202	0.1668	0.105*	

### Atomic displacement parameters (Å^2^)

**Table:**

	*U*^11^	*U*^22^	*U*^33^	*U*^12^	*U*^13^	*U*^23^
Co1	0.0217 (3)	0.0286 (3)	0.0338 (3)	0.0002 (2)	0.00614 (19)	−0.0021 (2)
N1	0.0347 (13)	0.0453 (15)	0.0303 (12)	−0.0069 (11)	0.0082 (10)	−0.0116 (11)
N2	0.0266 (12)	0.0330 (13)	0.0308 (11)	−0.0021 (10)	0.0062 (9)	−0.0046 (10)
N3	0.0299 (13)	0.0402 (14)	0.0542 (14)	−0.0011 (11)	0.0085 (11)	−0.0106 (12)
N4	0.0394 (14)	0.0436 (16)	0.0480 (14)	−0.0049 (12)	0.0015 (11)	0.0072 (12)
O1	0.0414 (12)	0.0723 (17)	0.0415 (11)	−0.0166 (11)	0.0190 (10)	−0.0120 (11)
O2	0.0405 (11)	0.0343 (11)	0.0261 (9)	−0.0047 (9)	0.0082 (8)	−0.0015 (8)
C1	0.0287 (14)	0.0389 (17)	0.0334 (14)	−0.0016 (13)	0.0036 (11)	−0.0003 (12)
C2	0.0295 (14)	0.0256 (14)	0.0281 (13)	−0.0009 (11)	0.0035 (11)	0.0019 (11)
C3	0.0297 (14)	0.0312 (15)	0.0306 (13)	−0.0040 (12)	0.0066 (11)	−0.0051 (11)
C4	0.0392 (16)	0.0277 (15)	0.0390 (15)	−0.0049 (13)	0.0019 (12)	−0.0058 (12)
C5	0.048 (2)	0.047 (2)	0.088 (3)	0.0023 (17)	0.0093 (19)	0.009 (2)
C6	0.075 (3)	0.048 (3)	0.116 (4)	0.015 (2)	−0.003 (3)	0.014 (2)
C7	0.114 (4)	0.034 (2)	0.066 (2)	−0.003 (2)	−0.001 (2)	0.0057 (19)
C8	0.109 (4)	0.042 (2)	0.057 (2)	−0.009 (2)	0.029 (2)	0.0037 (17)
C9	0.069 (2)	0.0373 (19)	0.0565 (19)	0.0007 (17)	0.0248 (17)	0.0010 (16)
C10	0.0281 (14)	0.0396 (17)	0.0314 (14)	−0.0046 (13)	0.0058 (11)	0.0019 (12)
C11	0.0349 (17)	0.066 (2)	0.0576 (19)	−0.0035 (17)	0.0022 (14)	−0.0179 (17)
C12	0.0347 (19)	0.107 (3)	0.062 (2)	−0.011 (2)	−0.0046 (16)	−0.019 (2)
C13	0.0327 (18)	0.110 (4)	0.062 (2)	0.006 (2)	−0.0013 (17)	0.013 (2)
C14	0.047 (2)	0.063 (2)	0.075 (2)	0.0152 (19)	0.0041 (18)	0.007 (2)
C15	0.0419 (18)	0.047 (2)	0.0531 (18)	0.0017 (16)	−0.0014 (14)	−0.0023 (16)
C16	0.079 (3)	0.077 (3)	0.106 (4)	0.046 (3)	0.026 (3)	0.009 (3)
C17	0.072 (3)	0.094 (4)	0.097 (3)	0.043 (3)	0.011 (2)	0.037 (3)

### Geometric parameters (Å, °)

**Table:**

Co1—N2^i^	2.180 (2)	C5—H5	0.9300
Co1—N2	2.180 (2)	C6—C7	1.377 (6)
Co1—N3	2.123 (2)	C6—H6	0.9300
Co1—N3^i^	2.123 (2)	C7—C8	1.365 (6)
Co1—N4^i^	2.171 (2)	C7—H7	0.9300
Co1—N4	2.171 (2)	C8—C9	1.384 (5)
N1—C1	1.347 (3)	C8—H8	0.9300
N1—C3	1.452 (3)	C9—H9	0.9300
N1—H1	0.8600	C10—C11	1.372 (4)
N2—C2	1.346 (3)	C10—C15	1.378 (4)
N2—C1	1.397 (3)	C11—C12	1.386 (5)
N3—C16	1.444 (5)	C11—H11	0.9300
N3—H3A	0.9000	C12—C13	1.362 (6)
N3—H3B	0.9000	C12—H12	0.9300
N4—C17	1.452 (5)	C13—C14	1.359 (5)
N4—H4A	0.9000	C13—H13	0.9300
N4—H4B	0.9000	C14—C15	1.389 (5)
O1—C1	1.232 (3)	C14—H14	0.9300
O2—C2	1.239 (3)	C15—H15	0.9300
C2—C3	1.540 (4)	C16—C17	1.439 (6)
C3—C4	1.530 (4)	C16—H16A	0.9700
C3—C10	1.533 (4)	C16—H16B	0.9700
C4—C5	1.371 (4)	C17—H17A	0.9700
C4—C9	1.383 (4)	C17—H17B	0.9700
C5—C6	1.374 (5)		
			
N3—Co1—N3^i^	180.00 (17)	C9—C4—C3	120.4 (3)
N3—Co1—N4^i^	98.26 (10)	C4—C5—C6	121.5 (4)
N3^i^—Co1—N4^i^	81.74 (10)	C4—C5—H5	119.2
N3—Co1—N4	81.74 (10)	C6—C5—H5	119.2
N3^i^—Co1—N4	98.26 (10)	C5—C6—C7	119.8 (4)
N4^i^—Co1—N4	180.00 (13)	C5—C6—H6	120.1
N3—Co1—N2^i^	89.15 (9)	C7—C6—H6	120.1
N3^i^—Co1—N2^i^	90.85 (9)	C8—C7—C6	120.0 (4)
N4^i^—Co1—N2^i^	87.67 (9)	C8—C7—H7	120.0
N4—Co1—N2^i^	92.33 (9)	C6—C7—H7	120.0
N3—Co1—N2	90.85 (9)	C7—C8—C9	119.6 (4)
N3^i^—Co1—N2	89.15 (9)	C7—C8—H8	120.2
N4^i^—Co1—N2	92.33 (9)	C9—C8—H8	120.2
N4—Co1—N2	87.67 (9)	C4—C9—C8	121.2 (4)
N2^i^—Co1—N2	180.00 (13)	C4—C9—H9	119.4
C1—N1—C3	111.7 (2)	C8—C9—H9	119.4
C1—N1—H1	124.2	C11—C10—C15	118.2 (3)
C3—N1—H1	124.2	C11—C10—C3	118.2 (3)
C2—N2—C1	107.1 (2)	C15—C10—C3	123.5 (3)
C2—N2—Co1	125.97 (16)	C10—C11—C12	120.9 (4)
C1—N2—Co1	126.87 (17)	C10—C11—H11	119.5
C16—N3—Co1	108.4 (2)	C12—C11—H11	119.5
C16—N3—H3A	110.0	C13—C12—C11	120.2 (4)
Co1—N3—H3A	110.0	C13—C12—H12	119.9
C16—N3—H3B	110.0	C11—C12—H12	119.9
Co1—N3—H3B	110.0	C14—C13—C12	119.8 (3)
H3A—N3—H3B	108.4	C14—C13—H13	120.1
C17—N4—Co1	106.7 (2)	C12—C13—H13	120.1
C17—N4—H4A	110.4	C13—C14—C15	120.2 (4)
Co1—N4—H4A	110.4	C13—C14—H14	119.9
C17—N4—H4B	110.4	C15—C14—H14	119.9
Co1—N4—H4B	110.4	C10—C15—C14	120.6 (3)
H4A—N4—H4B	108.6	C10—C15—H15	119.7
O1—C1—N1	124.4 (3)	C14—C15—H15	119.7
O1—C1—N2	124.6 (3)	C17—C16—N3	114.5 (3)
N1—C1—N2	111.0 (2)	C17—C16—H16A	108.6
O2—C2—N2	126.1 (2)	N3—C16—H16A	108.6
O2—C2—C3	122.7 (2)	C17—C16—H16B	108.6
N2—C2—C3	111.2 (2)	N3—C16—H16B	108.6
N1—C3—C4	111.7 (2)	H16A—C16—H16B	107.6
N1—C3—C10	110.4 (2)	C16—C17—N4	113.1 (3)
C4—C3—C10	112.6 (2)	C16—C17—H17A	109.0
N1—C3—C2	99.0 (2)	N4—C17—H17A	109.0
C4—C3—C2	108.7 (2)	C16—C17—H17B	109.0
C10—C3—C2	113.6 (2)	N4—C17—H17B	109.0
C5—C4—C9	117.9 (3)	H17A—C17—H17B	107.8
C5—C4—C3	121.6 (3)		
			
N3—Co1—N2—C2	36.0 (2)	O2—C2—C3—C4	63.0 (3)
N3^i^—Co1—N2—C2	−144.0 (2)	N2—C2—C3—C4	−115.9 (2)
N4^i^—Co1—N2—C2	−62.3 (2)	O2—C2—C3—C10	−63.3 (3)
N4—Co1—N2—C2	117.7 (2)	N2—C2—C3—C10	117.7 (2)
N2^i^—Co1—N2—C2	−164 (100)	N1—C3—C4—C5	−22.4 (4)
N3—Co1—N2—C1	−142.2 (2)	C10—C3—C4—C5	−147.3 (3)
N3^i^—Co1—N2—C1	37.8 (2)	C2—C3—C4—C5	85.8 (3)
N4^i^—Co1—N2—C1	119.5 (2)	N1—C3—C4—C9	160.3 (3)
N4—Co1—N2—C1	−60.5 (2)	C10—C3—C4—C9	35.4 (4)
N2^i^—Co1—N2—C1	18 (100)	C2—C3—C4—C9	−91.5 (3)
N3^i^—Co1—N3—C16	63 (100)	C9—C4—C5—C6	1.0 (5)
N4^i^—Co1—N3—C16	−170.8 (3)	C3—C4—C5—C6	−176.4 (3)
N4—Co1—N3—C16	9.2 (3)	C4—C5—C6—C7	−0.5 (7)
N2^i^—Co1—N3—C16	−83.3 (3)	C5—C6—C7—C8	0.0 (7)
N2—Co1—N3—C16	96.7 (3)	C6—C7—C8—C9	−0.2 (6)
N3—Co1—N4—C17	13.2 (3)	C5—C4—C9—C8	−1.2 (5)
N3^i^—Co1—N4—C17	−166.8 (3)	C3—C4—C9—C8	176.2 (3)
N4^i^—Co1—N4—C17	−140 (100)	C7—C8—C9—C4	0.8 (6)
N2^i^—Co1—N4—C17	102.0 (3)	N1—C3—C10—C11	−65.3 (3)
N2—Co1—N4—C17	−78.0 (3)	C4—C3—C10—C11	60.3 (3)
C3—N1—C1—O1	−178.6 (3)	C2—C3—C10—C11	−175.5 (3)
C3—N1—C1—N2	−0.7 (3)	N1—C3—C10—C15	111.6 (3)
C2—N2—C1—O1	179.1 (3)	C4—C3—C10—C15	−122.8 (3)
Co1—N2—C1—O1	−2.4 (4)	C2—C3—C10—C15	1.5 (4)
C2—N2—C1—N1	1.1 (3)	C15—C10—C11—C12	−1.0 (5)
Co1—N2—C1—N1	179.62 (18)	C3—C10—C11—C12	176.2 (3)
C1—N2—C2—O2	180.0 (3)	C10—C11—C12—C13	0.2 (6)
Co1—N2—C2—O2	1.5 (4)	C11—C12—C13—C14	1.3 (6)
C1—N2—C2—C3	−1.1 (3)	C12—C13—C14—C15	−1.9 (6)
Co1—N2—C2—C3	−179.66 (16)	C11—C10—C15—C14	0.3 (5)
C1—N1—C3—C4	114.4 (3)	C3—C10—C15—C14	−176.6 (3)
C1—N1—C3—C10	−119.5 (3)	C13—C14—C15—C10	1.1 (5)
C1—N1—C3—C2	0.0 (3)	Co1—N3—C16—C17	−31.8 (5)
O2—C2—C3—N1	179.7 (2)	N3—C16—C17—N4	46.5 (6)
N2—C2—C3—N1	0.7 (3)	Co1—N4—C17—C16	−34.6 (5)

Symmetry codes: (i) −*x*+1, −*y*, −*z*.

### Hydrogen-bond geometry (Å, °)

**Table:**

*D*—H···*A*	*D*—H	H···*A*	*D*···*A*	*D*—H···*A*
N1—H1···O2^ii^	0.86	2.00	2.861 (3)	173
N3—H3A···O1^i^	0.90	2.18	2.947 (3)	143
N3—H3B···O2	0.90	2.20	2.966 (3)	143

Symmetry codes: (ii) *x*, −*y*+1/2, *z*+1/2; (i) −*x*+1, −*y*, −*z*.
